# Increased Mortality in Groups of Cattle Administered the β-Adrenergic Agonists Ractopamine Hydrochloride and Zilpaterol Hydrochloride

**DOI:** 10.1371/journal.pone.0091177

**Published:** 2014-03-12

**Authors:** Guy H. Loneragan, Daniel U. Thomson, H. Morgan Scott

**Affiliations:** 1 International Center for Food Industry Excellence, Department of Animal and Food Sciences, College of Agriculture and Natural Resources, Texas Tech University, Lubbock, Texas, United States of America; 2 Department of Clinical Sciences, College of Veterinary Medicine, Kansas State University, Manhattan, Kansas, United States of America; 3 Department of Diagnostic Medicine/Pathobiology, College of Veterinary Medicine, Kansas State University, Manhattan, Kansas, United States of America; CSIRO, Australia

## Abstract

The United States Food and Drug Administration (FDA) approved two β-adrenergic agonists (βAA) for in-feed administration to cattle fed in confinement for human consumption. Anecdotal reports have generated concern that administration of βAA might be associated with an increased incidence of cattle deaths. Our objectives, therefore, were to a) quantify the association between βAA administration and mortality in feedlot cattle, and b) explore those variables that may confound or modify this association. Three datasets were acquired for analysis: one included information from randomized and controlled clinical trials of the βAA ractopamine hydrochloride, while the other two were observational data on zilpaterol hydrochloride administration to large numbers of cattle housed, fed, and cared for using routine commercial production practices in the U.S. Various population and time at-risk models were developed to explore potential βAA relationships with mortality, as well as the extent of confounding and effect modification. Measures of effect were relatively consistent across datasets and models in that the cumulative risk and incidence rate of death was 75 to 90% greater in animals administered the βAA compared to contemporaneous controls. During the exposure period, 40 to 50% of deaths among groups administered the βAA were attributed to administration of the drug. None of the available covariates meaningfully confounded the relationship between βAA and increased mortality. Only month of slaughter, presumably a proxy for climate, consistently modified the effect in that the biological association was generally greatest during the warmer months of the year. While death is a rare event in feedlot cattle, the data reported herein provide compelling evidence that mortality is nevertheless increased in response to administration of FDA-approved βAA and represents a heretofore unquantified adverse drug event.

## Introduction

The United States Food and Drug Administration (FDA) approved two β-adrenergic agonists (βAA) for in-feed administration to cattle that are fed in confinement (i.e., typically feedlot operations) for human consumption [1], [2]. When administered according to label directions, βAA result in well-characterized and predictable improvements in the rate and efficiency of weight gain, as well as increased leanness and yield of edible products derived from beef carcasses [3]–[7]. Ractopamine hydrochloride (RH) was the first βAA approved in cattle; further, RH may be used in a variety of dosages and has also been approved for administration to swine and turkeys. Ractopamine hydrochloride is included in cattle feed during the final 28 to 42 days of the fattening period. Zilpaterol hydrochloride (ZH), the second βAA approved by the FDA, may only be used at a single rate of inclusion in cattle feeds. It is included in cattle feed for 20 to 40 days prior to slaughter; however, in contrast to RH, a 3-day period during which ZH may not be administered must be observed prior to shipment to the abattoir (i.e., slaughter withholding).

In addition to their production uses in food-animal production, β-adrenergic agonists are routinely used in human clinical medicine for various conditions such as acute therapeutic intervention and maintenance care of asthma and chronic obstructive pulmonary disease (i.e., COPD). In human clinical medicine, these drugs are not innocuous in that side effects (or adverse unintended consequences) of approved medical uses of βAA have been observed and include an increased risk of asthma exacerbations and hospitalizations, arrhythmias, myocardial infarction, and death [8]–[12]. Unintended consequences have also been observed with βAA use in several animal species. For example, observations both in non-ruminant and ruminant species indicate that administration of βAA is associated, albeit somewhat inconsistently, with elevated heart rates, body temperature, physical activity, lameness or foot lesions, and aggression [13]–[18]. Given the widespread distribution of β-adrenoreceptors in the body, some of these unintended consequences, such as elevated heart rate, might (or ought to) be expected with βAA administration.

With some similarity to observations of increased risk of mortality associated with use of certain βAA in human clinical medicine [9], [19], unpublished reports from some end-users of RH and ZH indicated the potential for an increase in mortality in cattle associated with FDA-approved administration of βAA. Mortality in feedlot cattle represents a clear and meaningful economic loss to producers. Death loss also raises broader societal concerns about the welfare of animals fed βAA in that progression from a healthy status to death may include in pain and suffering in affected animals. If a relationship between βAA administration and increased risk of mortality exists, it ought to stimulate discussion of the pros and cons of the use of drugs approved purely to improve the efficiencies of production yet offering no offsetting health benefits to the animals. As an example, some antibiotics are believed to improve production efficiency, at least in part, by controlling or preventing subclinical disease. To our knowledge, no beneficial ‘side-effect’ favoring improved health or welfare, or control or prevention of disease in response to FDA-approved βAA administration in cattle is believed to exist. Our objectives, therefore, were to: a) quantify the association between βAA administration and mortality in feedlot cattle, and b) explore those variables that may confound or modify this association.

## Methods

Three confidential datasets were received either through solicitation by the first author in the case of RH or following requests for analytical support from the owners of the data (i.e., feedlot operators) in the case of ZH. The authors adhere to all the PLOS ONE policies on sharing data and materials. Different models were constructed and where possible and appropriate, a number of covariates (as potential and plausible confounders and effect modifiers) were evaluated. Datasets analyzed included both experimental trials (i.e., clinical trials involving randomized treatment allocation) and observational studies (i.e., treatment allocation was determined by factors other than a random process).

### Description of Datasets

The first dataset (hereafter ***4-company RH dataset***) included information concerning administration of RH. A convenience sample of cattle-feeding companies was contacted to evaluate whether or not they had performed field-based experiments of RH administration and, if so, to ascertain their willingness to provide their data for further analysis. Inclusion criteria included: a) experimental observations of RH administered to cattle pens according to label directions so as to provide a target dose of 200 mg/animal/day for the 28 to 42 days immediately prior to shipment to the abattoir, b) inclusion of appropriate contemporaneous control pens, and c) randomization performed either at the group level, or at the individual level. Groups were then classified as either unexposed in that all animals within the group were fed the usual fattening diet, or else as exposed in that all animals within the group were fed the usual fattening diet with RH incorporated to provide the target dose.

This first aggregated dataset included information from four companies and collectively included 12 separate randomized experiments. All experiments used a randomized block design in that there were at least 2 groups per block, i.e., one administered RH and the other not. These data included information suitable for analysis on a total of 79,171 cattle. These animals were aggregated into 509 groups that averaged 155.5 (standard deviation [SD] = 70.9, minimum [MIN] = 42, maximum [MAX] = 381) animals per group. The number of studies and groups varied by company in that company A provided data on 1,510 animals that were enrolled in 1 study and housed in 24 groups, company B provided data on 5,696 animals across 3 studies and housed in 52 groups, company C provided data on 62,379 animals across 3 studies and housed in 329 groups, and company D provided data on 9,586 animals across 5 studies and housed in a total of 104 groups. The combined 6 studies conducted by companies B and C were conducted in 6 different feedlots whereas the 5 studies of company D were all conducted in the same feedlot. The vast majority of cattle were steers (i.e., castrated males; n = 72,868 in 441 groups) with the balance being females (i.e., heifers; n = 6,303 in 68 groups). Heifers were exclusively enrolled as the study population in 3 of the 5 experiments of company D.

The second dataset (***multi-feedlot ZH dataset***) concerned administration of ZH and included observational data from nine feedlots. Data on 722,704 animals were provided and the cattle were housed in 3,110 groups of an average size of 232.4 animals per group (SD = 91.3, MIN = 32, MAX = 943). Out of these animals, 79.3% (n = 573,076) were steers and 20.7% (n = 149,628) were heifers. The at-risk period of interest for exposed animals included both the period during which ZH was administered as well as the slaughter-withholding period. This slaughter-withholding period must be a minimum of 3 days but may be longer depending on various marketing strategies used by the feedlots. For the unexposed population in the multi-feedlot ZH dataset, the at-risk period was calculated by including the typical number of days ZH was administered to the exposed cohort and the typical slaughter-withholding period. Based on the observed and calculated at-risk periods for the exposed and unexposed cohorts, respectively, the mean at-risk periods consisted of the final 29.4 and 29.2 days prior to shipment, respectively. The data were unbalanced in that there were 2,775 groups comprised of 637,339 animals administered ZH (i.e., exposed cohort) and 335 groups comprised of 85,365 animals that served as comparative controls (i.e., unexposed cohort). The mean group sizes were 229.7 (SD = 90.7, MIN = 32, MAX = 943) and 254 (SD = 94.0, MIN = 55, MAX = 610), respectively. The number of animals per feedlot for which data were provided varied from 61,059 to 123,679. Heifers were represented in the data from 8 of the 9 feedlots.

The third dataset (***single-feedlot ZH dataset***) included observational data on 149,636 animals that were housed in 835 groups in a single feedlot. Of the population at risk, 88.7% (n = 132,725) were steers and 11.3% (n = 16,911) were heifers. The data were more balanced than the multi-feedlot ZH dataset in that 56.1% of the cattle (n = 83,865 in 470 groups) were administered ZH (i.e., exposed cohort) whereas 43.9% of the cattle (n = 65,711 in 365 groups) served as the contemporaneous control cohort. The mean group sizes were 178.4 (SD = 76.3, MIN = 30, MAX = 352) and 180.2 (SD = 63.9, MIN = 54, MAX = 382), respectively. The feedlot was managed in such a way that ZH was administered 21 days prior to shipment and a 3-day withdrawal period was observed. This 24-day period was considered the at-risk period. Consequently, the final 24 days prior to shipment were considered the comparative at-risk period for those animals not administered ZH.

### Data Analyses

The primary outcome variable of interest across all datasets was the number of animals that died in each group during the at-risk period (consequently, the group may be considered the experimental unit of interest). This outcome variable, therefore, represents a count response and the approaches described herein to model count data within groups in which the outcome may be clustered have been described [20]–[23]. Two offset variables were used as denominators in the various statistical models. The first was the natural logarithm of the population within a group (i.e., the at-risk population) at the start of the exposure period; the use of this offset allows calculation of model-adjusted estimates of the proportion the population at risk that died within each cohort [20]. Where the at-risk period varied across groups, i.e., in the 4-company RH and multi-feedlot ZH datasets, the natural logarithm of time at-risk was used (in other words, the total number of cattle-days within a group during the period of interest); the use of this offset allows calculation of model-adjusted estimates of the incidence of death (i.e., deaths per unit time) within each cohort [20]. Because information on the day during the at-risk period that individual animals died was only available for Company C in the RH dataset, a uniform approach to estimating time at risk (expressed as cattle-days) was used for the 4-company RH and multi-feedlot ZH datasets. Time at risk was estimated as a function of the at-risk period (days) multiplied by the population at risk. To account for withdrawals due to death, half of the total possible days at-risk was subtracted from the group’s time at risk for each animal that died within the group.

Consistent across all datasets, therefore, were deaths within a group, whether they were administered a βAA or not, and population at risk. In addition, for the 4-company RH and multi-feedlot ZH datasets, time at risk was calculated using a common approach. Generalized linear mixed models were constructed using commercially available statistical analysis software (SAS System for Windows release 9.3, SAS Institute, Cary, NC) and parameterized similarly across datasets to account for the hierarchical nature of the data. A Poisson distribution was used with a log-link function. In all models, a group-level term was forced into the model to account for potential over-dispersion of the data (i.e., extra-Poisson variation). In the model of the 4-company RH dataset, random intercept terms were included for company, study within company, and block within study. In the multi-feedlot ZH dataset, a random intercept term was included to account for potential clustering of the outcome within feedlots. To explore potential modification of βAA effect on death loss across companies in the 4-company RH dataset and across feedlots in the multi-feedlot ZH dataset, the highest-level random-intercept term was changed from a random effect to a fixed effect to explore the interaction with exposure. Because of model convergence issues due to sparsely populated cells in the former dataset, when evaluating the interaction of feedlot and RH administration, data from company A was dropped from the model (i.e., n = 1,510 animals in 24 groups where no deaths were reported in either cohort).

Covariates were variably recorded across the three datasets. For example, within the 4-company RH dataset, the number of deaths within a group prior to exposure was relatively consistently recorded whereas month of shipment was only recorded for Company C. Further, in the multi-feedlot ZH dataset, a variety of covariates were consistently recorded and included: sex of the animals within a group, percentage of a group that died prior to the at-risk period, percentage of a group that were treated prior to the at-risk period, percentage of cattle within a group that had a predominantly black hide, mean carcass weight of the surviving animals that were shipped to slaughter, and month in which the at-risk period ended (i.e., animals were shipped to an abattoir for slaughter). However, in the single-feedlot ZH dataset only sex of the animal and the month in which the animals were shipped to an abattoir for slaughter were available for analysis. For each dataset, therefore, covariates that could potentially confound or modify the association between βAA use and death loss were evaluated in univariate models to test their association with mortality. Model structures were similar to those described above. Covariates with *P* values less than or equal to 0.20 were included as fixed effects in multivariable models that included main effects (i.e., potential confounders) and terms for the interaction of each of these main effects with exposure to βAA (i.e., effect-measure modifiers). While maintaining hierarchy within variables, terms were removed from the model in a backward, stepwise manner using α = 0.10 level of significance for retention. For all models, measures of burden (proportion of population at risk and incidence of mortality per time at risk) and measures of effect (cumulative relative risk [RR] and incidence rate ratio [IRR], respectively) were computed from the model estimates and presented with their 95% confidence limits (CL) and *P* values where appropriate.

In addition to the primary outcome of mortality, secondary outcomes were available for both the multi-feedlot ZH and single-feedlot ZH datasets. Secondary count-based outcomes included number of animals treated for illness during the at-risk period and the number of carcasses that were classified as *dark, firm and dry* (which is colloquially referred to as *dark cutter* in the beef cattle industry). In addition, the proportion of cattle that could not be shipped to slaughter because they were within a slaughter-withholding period at the time the rest of the group was shipped was available in the single-feedlot dataset. This so-called *medicine hold* results from administration of a therapeutic drug, such as an injectable antimicrobial drug to treat bacterial bronchopneumonia; most such FDA-approved antimicrobial drugs have slaughter-withholding periods and these must be observed prior to shipment to slaughter for human consumption.

To explore the extent of unexplained model variation attributable to the levels of company and study within company, a multi-level, hierarchical model was constructed for the 4-company RH dataset using commercially available software (MLwiN 2.26, Centre for Multilevel Modelling, University of Bristol, Bristol, UK). Four levels of organization were included in the model: company, study within company, block within study, and group within block; generalized linear mixed models within a Poisson distribution were constructed. The dependent and offset variables were those described above and RH administration was the independent variable of interest. After accounting for RH administration as a fixed effect in the model, unexplained variation was partitioned to the highest 3 level terms (i.e., level 1 variance was not calculated given the assumptions of the Poisson model) [22]. Model estimation was performed using reweighted iterative generalized least squares and 2^nd^-order penalized quasi-likelihood approximation, while allowing for over-dispersion of the data [24].

Multivariable semi-parametric survival analysis was performed on the data provided by Company C owing to the rich level of detail available (Stata version 12.1, Stata Corp., College Station, TX). The multivariable model accounted for fixed effects of study, month of shipment to slaughter, prior mortality events experienced within the cohort, and exposure to RH. In addition, the model accounted for the shared frailty experience of animals within each group. Backwards elimination was employed to yield the final model (accounting for potential confounding variables and effect modifiers) and the importance of the shared group frailty was determined using the parameter Theta.

## Results

### 4-company RH Dataset

Overall, 0.27% (n = 211) of the 79,171 cattle died during the at-risk period (Table 1). After accounting for various levels of clustering, the estimates of cumulative risk and the incidence rate of death were 0.26% (95% confidence limits [CL] = 0.16, 0.40) and 0.86 (95% CL = 0.58, 1.30) deaths per 10,000 cattle days, respectively. Model-adjusted estimates of risk and incidence of death for cattle administered RH were 0.34% (95% CL = 0.25, 0.45) and 1.12 (95% CL = 0.85, 1.45) deaths per 10,000 cattle days at risk, respectively. For cattle not administered RH, risk and incidence of death were 0.18% (95% CL = 0.13, 0.25) and 0.59 (95% CL = 0.43, 0.83) deaths per 10,000 cattle days, respectively.

**Table 1:** Summary statistics for death loss (counts, and crude and model-adjusted estimates) by dataset for all cattle, the exposed cohort (i.e., groups of cattle administered either ractopamine hydrochloride [RH] or zilpaterol hydrochloride [ZH]), and the unexposed cohort.

		Statistic						
Dataset	Cohort	Populationat risk (n)	Deaths (n)	Crude estimate ofcumulative risk (%)	Model-adjustedcumulative risk (%)	95% confidencelimits	Model-adjustedincidence (deaths/10,000 animal days)	95% confidencelimits
4-companyRH dataset	All cattle	79,171	211	0.27	0.26	0.16, 0.40	0.86	0.58, 1.30
	Exposed	39,890	139	0.35	0.34	0.25, 0.41	1.12	0.85, 1.45
	Unexposed	39,281	72	0.18	0.18	0.13, 0.25	0.59	0.43, 0.83
Multi-feedlotZH dataset	All cattle	722,704	3,657	0.51	0.50	0.44, 0.57	1.68	1.51, 1.86
	Exposed	637,339	3,405	0.53	0.53	0.44, 0.59	1.77	1.62, 1.92
	Unexposed	85,365	252	0.30	0.30	0.25, 0.36	1.01	0.85, 1.19
Single-feedlotZH dataset	All cattle	149,636	571	0.38	0.38	0.35, 0.42	*	*
	Exposed	83,865	401	0.48	0.48	0.43, 0.43	*	*
	Unexposed	65,771	170	0.26	0.26	0.22, 0.31	*	*

* Not calculated as both cohorts had the same exposure period of 24 days.

**Figure 1:**
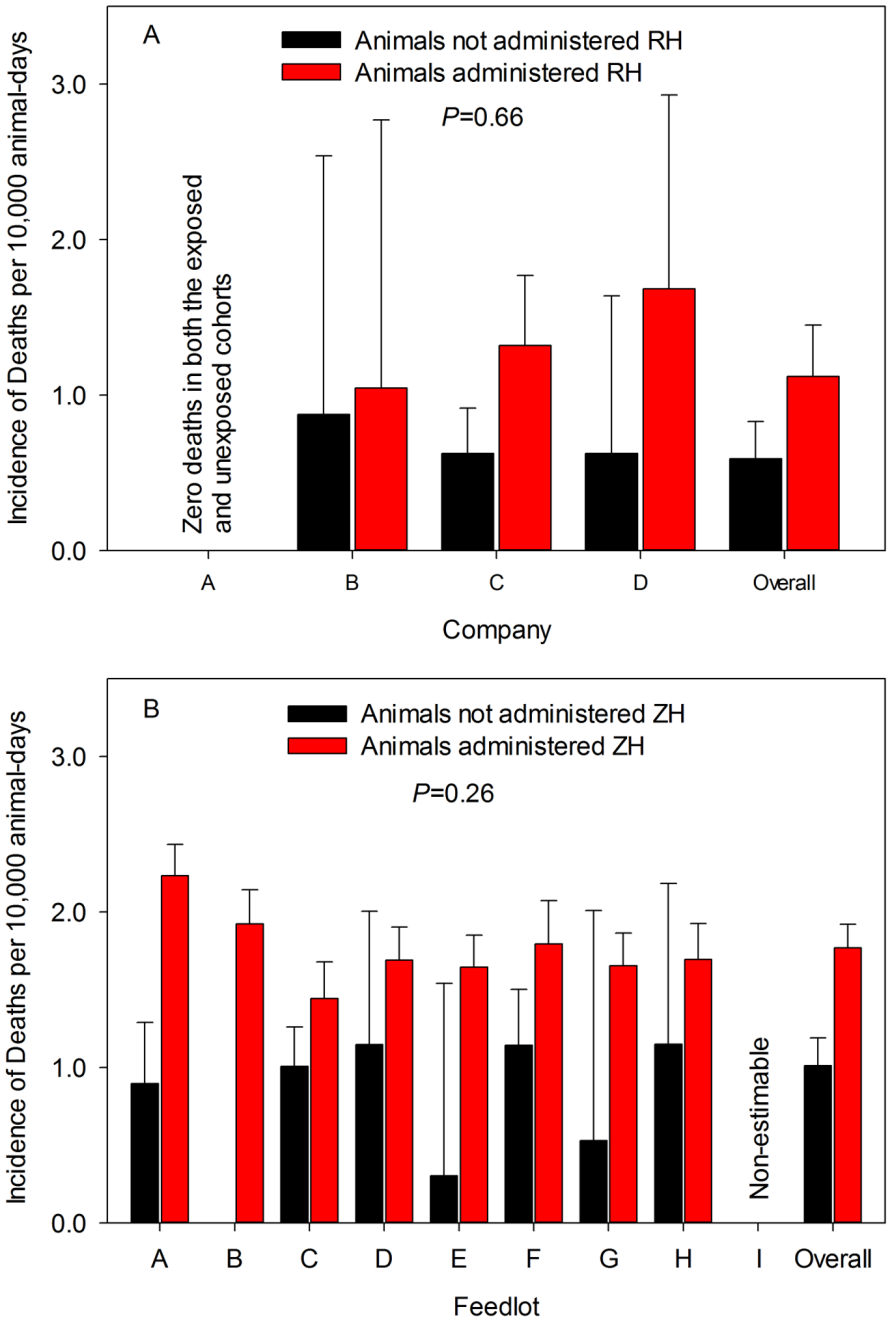
Association between β-adrenergic agonist administration and mortality. Model-adjusted estimates of incidence of death per 10,000 animal-days for cattle administered either ractopamine hydrochloride (RH – graph A) or zilpaterol hydrochloride (ZH – graph B) compared to a diet without a beta agonist. No deaths were reported for Company A (graph A) and rates for Feedlot I (graph B) were non-estimable. *P* values are those associated with interaction term for exposure by company (graph A) or feedlot (graph B). Bars represent upper 95% confidence limit.

After controlling for clustering within company, study, block and group, cattle administered RH were 91% more likely to die than control animals during the at-risk period (RR = 1.90 [95% CL = 1.38, 2.60]; *P*<0.01). A very similar measure of effect was observed for the incidence rate ratio (IRR = 1.90 [95% CL = 1.38, 2.61]; *P*<0.01). The only potential covariate collected across multiple studies was the number of animals that died in each group prior to the at-risk period. This variable neither modified the effect of exposure on mortality (*P* = 0.77) nor did it confound the association between βAA and increased death loss (*P* = 0.63). There was no evidence that the association between RH administration and increased death loss varied across the 3 companies that supplied data in which at least one death was observed (*P* = 0.66; Figure 1). Moreover, in the multilevel hierarchical model that included all 4 companies, there was no unexplained variation attributed to unmeasured factors across companies (model variance = 0.00 [SE = 0.00]). At lower levels of organization, the unexplained model variation was 4-fold greater among blocks within studies (model variance = 0.169 [SE = 0.120]) than among studies within companies (model variance = 0.048 [SE = 0.072]).

**Figure 2:**
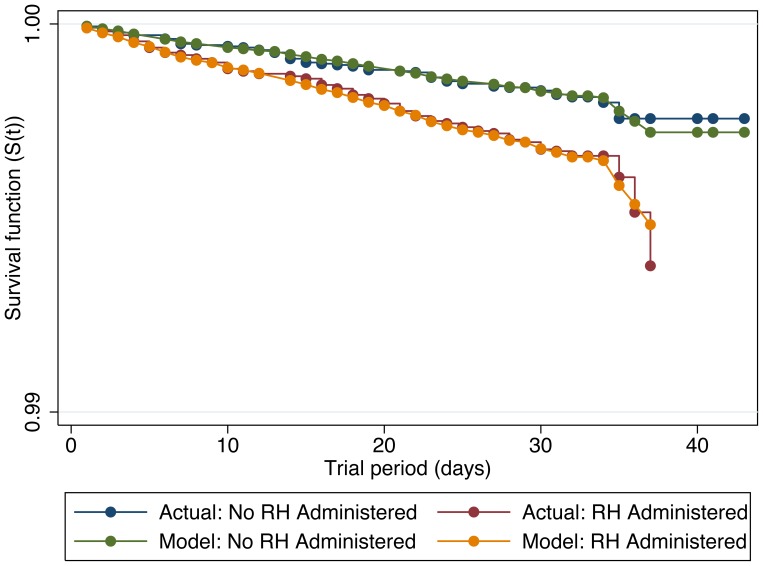
Survival analysis for cattle administered a β-adrenergic agonist. Kaplan-Meier non-parametric (actual) and Cox proportional hazards (predicted) survivor functions (S(t)) for cattle administered a diet containing ractopamine hydrochloride (RH) compared to a diet without RH in Company C.

**Figure 3:**
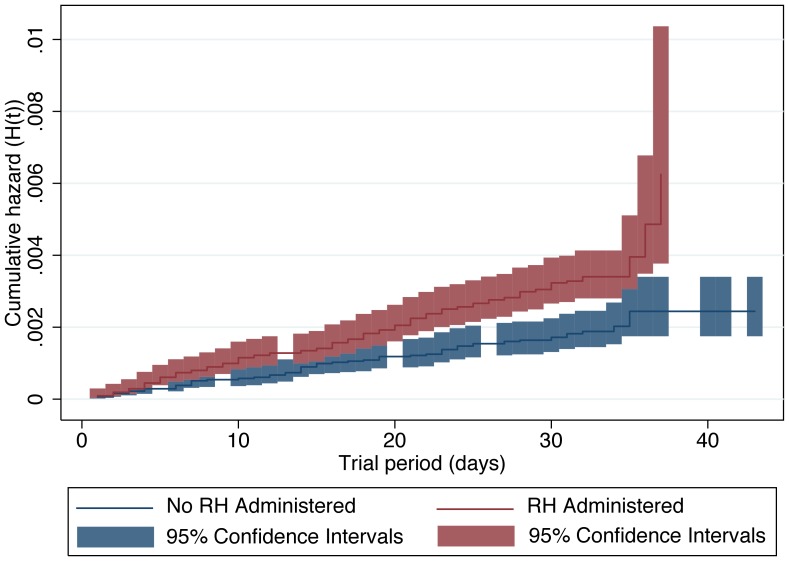
Force of mortality among cattle administered a β-adrenergic agonist. Empirical cumulative hazard function (H(t)) and 95% confidence intervals (during those time periods where mortalities occurred) for cattle administered a diet containing ractopamine hydrochloride (RH) compared to a diet without RH in Company C.

Company C provided information on 167 deaths across 1,983,564 animal-days and within this company, the incidence of death was 83% greater among those animals administered RH compared to controls (IRR = 1.83; 95% CL = 1.32, 2.56; *P*<0.01). A graphical representation of the unadjusted Kaplan-Meier non-parametric survival experiences for each set of treatment cohorts is presented in Figure 2. The smoothed instantaneous force of mortality for each cohort set (i.e., the hazard function [h(t)]) is presented in Figure 3 and suggests that the constant hazard assumption was met for approximately the first 25–28 days of the at risk period. Moreover, the proportional hazards assumption was met throughout the entire at-risk period. In addition, the pen-level mortality rate prior to exposure was evaluated as a covariate but was neither associated with the incidence of death during exposure (*P* = 0.61), nor did this variable confound the relation between exposure to RH and survivor function. Importantly, the multivariable adjusted treatment hazard ratio (HR) of 2.00 (95% CL = 1.36, 2.96; *P*<0.01) was greater than that observed when the other significant covariates (i.e., fixed effects of feedlot and month of slaughter) and the shared frailties of group were ignored. Because the force of mortality (hazard) was relatively constant over time, the HR is comparable to IRR; the observed IRR was 1.83 and was included within the 95% confidence interval of the HR.

### Multi-feedlot ZH Dataset

Of the 722,704 cattle at risk, 0.51% (n = 3,657) of animals died during the exposure period (Table 1). After accounting for feedlot- and group-level clustering, the estimates of risk and incidence of death were 0.50% (95% CL = 0.44, 0.57) and 1.68 (95% CL = 1.51, 1.86) deaths per 10,000 animal-days, respectively. Model-adjusted estimates of the risk and incidence of death for cattle administered ZH were 0.53% (95% CL = 0.47, 0.59) and 1.77 (95% CL = 1.62, 1.92) deaths per 10,000 animal-days, respectively. Model-adjusted estimates among the unexposed cohort were 0.30 (95% CL = 0.25, 0.36) and 1.01 (95% CL = 0.85, 1.19) deaths per 10,000 animal-days, respectively. After adjusting for the various levels of clustering, administration of ZH was associated with a significant increase in the likelihood of death (RR = 1.76 [95% CL = 1.50, 2.05]; *P*<0.01). When time at risk during the exposure period was used as the offset variable instead of population at risk, the measure of effect was similar in that the IRR was 1.75 (95% CL = 1.50, 2.05; *P*<0.01).

Covariates included in the multivariable model as main effects (i.e., potential confounders) along with terms representing their interactions with exposure to ZH (i.e., effect modifiers) included (Table 2):

**Table 2:** Covariates evaluated for an association with mortality in groups of cattle either administered zilpaterol hydrochloride (ZH) or not in the multi-feedlot ZH dataset in univariate and multivariable models.

		Univariate model		Multivariable model			
Measure of mortalityin groups of cattle	Covariate	*P* value	Included inmultivariatemodel	Main effect*P* value*	Retained infinal model	*P* value* ofinteractionterm**	Retained infinal model
Cumulative risk (%)	Sex of the animals within agroup	<0.01	Yes	<0.01	Yes	0.46	No
	Percentage of a group thatdied prior to the at-risk period	0.13	Yes	0.31	No	0.39	No
	Percentage of a group thatwere treated prior to the at-risk period	<0.01	Yes	<0.01	Yes	0.41	No
	Percentage of cattle within a group thathad a predominantly black hide	<0.01	Yes	<0.01	Yes	0.66	No
	Mean carcass weight of the survivinganimals that were shipped to slaughter	<0.01	Yes	0.77	No	0.73	No
	Month in which the at-riskperiod ended	<0.01	Yes	<0.01	Yes	0.07	Yes
	Days at the feedlot prior to exposure	0.23	No	–	–	–	–
Incidence (deaths/10,000animal-days)	Sex of the animals within agroup	<0.01	Yes	<0.01	Yes	0.42	No
	Percentage of a group thatdied prior to the at-risk period	0.05	Yes	0.70	No	0.49	No
	Percentage of a group that were treated prior tothe at-risk period	<0.01	Yes	<0.01	Yes	0.50	No
	Percentage of cattle within a group thathad a predominantly black hide	<0.01	Yes	<0.01	Yes	0.85	No
	Mean carcass weight of the survivinganimals that were shipped to slaughter	<0.01	Yes	0.50	No	0.95	No
	Month in which the at-riskperiod ended	<0.01	Yes	<0.01	Yes	0.14	No
	Days at the feedlot prior toexposure	0.91	No	–	–	–	–

Administration of ZH was associated with mortality (*P*<0.01) in all multivariable models.

*P value indicated was that observed at the time of removal from the multivariable model if >0.10, or if retained, i.e., ≤0.10, its value in the final multivariable model.

** Interaction term of the covariate with administration of ZH, i.e., evaluation of potential modification of the association between death loss and administration.

- Sex of the animals within a group,
- Percentage of a group that died prior to the at-risk period,
- Percentage of a group that were treated prior to the at-risk period,
- Percentage of cattle within a group that had a predominantly black hide,
- Mean carcass weight of the surviving animals that were shipped to slaughter, and
- Month in which the at-risk period ended (i.e., animals were shipped to an abattoir for slaughter).

**Figure 4:**
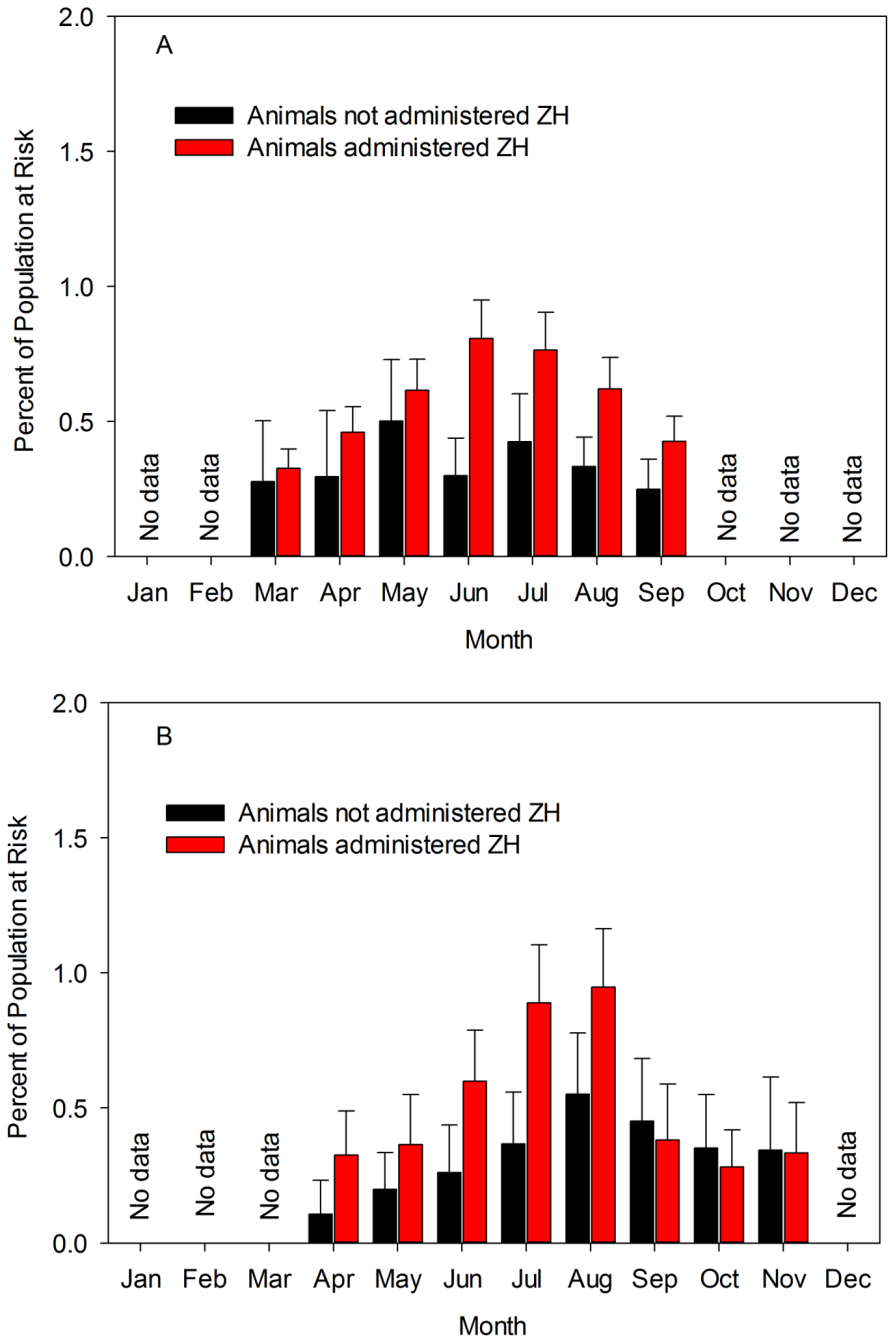
Seasonal modification of the association between β-adrenergic agonist administration and mortality. Model-adjusted estimates of the percentage of cattle that died among groups of animals administered zilpaterol hydrochloride (ZH) compared to a diet without ZH by month in which they were shipped to slaughter. Graph A represents 722,704 animals housed in 9 feedlots and graph B represents 149,636 animals housed in a single feedlot.

Of the main effect terms, only percentage of a group that died prior to the at-risk period and mean carcass weight of animals shipped to slaughter were removed from the final model. The only effect modifier (i.e., interaction term) that was retained was month in which the animals were shipped to the abattoir (*P* = 0.07). While controlling for the other covariates, RR estimates were 1.17 (95% CL = 0.64, 2.14; *P* = 0.60; Figure 4); 1.56 (95% CL = 0.86, 2.82; *P* = 0.15); 1.23 (95% CL = 0.87, 1.73; *P* = 0.24); 2.69 (95% CL = 1.86, 3.90; *P*<0.01); 1.80 (95% CL = 1.28, 2.53; *P*<0.01); 1.86 (95% CL = 1.42, 2.43; *P*<0.01) and 1.71 (95% CL = 1.18, 2.49; *P* = 0.01) for March, April, May, June, July, August, and September, respectively.

Averaged across the means of all other terms in the model, treatment remained significantly associated with mortality (RR = 1.66 [95% CL = 1.40, 1.96]; *P*<0.01). Thus, its effect did not appear to be confounded to any meaningful extent by other variables (those listed in bullet form above) in that the measure of effect from the multivariable model was similar to the reduced model in which only exposure was included as a fixed effect.

The same set of covariates listed above were associated with the incidence rate of death in univariate models; that is, the models in which when time at risk was included as the offset variable. These variables were subsequently included as covariates (main effects and effect modifiers) in the multivariable model of the incidence of death. While the same main effects were retained in the final model as those for the population at-risk model, no effect modifiers were retained in the final model (i.e., *P*>0.14 for all interaction terms). Controlling for the covariates that remained in the model (i.e., sex, month shipped to slaughter, percent of the group with a black hide, and percent of pen treated prior to the at-risk period), the incidence of death was 80% greater in animals administered ZH than the comparative control cohort (IRR = 1.80 [95% CL = 1.55, 2.10]; *P*<0.01). The association between incidence rate of death and administration of βAA, therefore, did not appear to be confounded by the covariates available for analysis.

There was little evidence of feedlot-to-feedlot variation in the association between ZH and increased risk of death. In the population at-risk model, for example, the covariance parameter was much greater at the group-level (1.14 [SE = 0.03]) than that observed at the feedlot-level (0.04 [SE = 0.02]). In the alternative model that treated feedlot and its interaction with exposure as ZH fixed effects, there was no evidence that the association between administration of ZH and increased mortality was modified by which feedlot the animals were housed in either the population at risk (*P* = 0.16) or time at risk models (*P* = 0.26; Figure 1).

Two secondary outcomes were available for analysis (Table 3). Cattle administered ZH were 33% more likely (RR = 1.33 [95% CL = 1.18, 1.50]; *P*<0.01) to require treatment for illness during the at-risk period than animals not administered ZH. In addition, an association between ZH administration and an increased likelihood of the animal’s beef being classified as *dark, firm and dry* was detected (Table 4); however, this latter association was modified by the sex of the animal (*P* = 0.01). The carcasses derived from steers administered ZH were 2.31 times more likely to be classified as *dark, firm and dry* compared to carcasses of steers not administered ZH (RR = 2.31 [95% CL = 1.77, 3.02]; *P*<0.01). Of the steer carcasses, 1.87 and 0.81%, of those administered ZH and the unexposed cohort, respectively, were classified as *dark, firm and dry*. No such association was observed with treatment among the carcasses derived from heifers (*P* = 0.36); of the heifer carcasses, 1.30 and 1.07%, of those administered ZH and the unexposed cohort, respectively, were classified as *dark, firm and dry*.

**Table 3:** Cumulative risk of treatment for disease among the exposed cohort (i.e., groups of cattle administered zilpaterol hydrochloride [ZH]) and the unexposed cohort.

			Statistic				
Dataset	Disease grouping	Cohort	Cumulative risk (%)	95% confidence limits	Relative risk	95% confidence limits	*P* value
Multi-feedlot ZH dataset	All diseases	Exposed	1.35	1.13, 1.62	1.33	1.18, 1.50	<0.01
	All diseases	Unexposed	1.01	0.83, 1.24	–	–	–
Single-feedlot ZH dataset	All diseases	Exposed	1.33	1.22, 1.46	1.23	1.07, 1.41	<0.01
	All diseases	Unexposed	1.09	0.98, 1.21	–	–	–
	Respiratory	Exposed	0.86	0.78, 0.95	2.31	0.55, 0.79	<0.01
	Respiratory	Unexposed	0.37	0.31, 0.44	–	–	–
	Digestive and other	Exposed	0.47	0.42, 0.54	0.66	0.55, 0.79	<0.01
	Digestive and other	Unexposed	0.72	0.63, 0.81	–	–	–

**Table 4:** Prevalence of carcasses classified as *dry, dark, and firm* among the exposed cohort (i.e., groups of cattle administered zilpaterol hydrochloride [ZH]) and the unexposed cohort.

		Statistic							
Dataset	Cohort	Prevalence(%)	95%confidencelimits	*P* valueassociatedwithmain effectcomparison	*P* valueassociated withinteractionwith sex	Prevalenceamong steers(%)	95%confidencelimits	Prevalenceamong heifers(%)	95%confidencelimits
Multi-feedlotZH dataset	Exposed	1.75	1.11, 2.75	<0.01	0.01	1.87	1.19, 2.95	1.30	0.82, 2.05
	Unexposed	0.86	0.53, 1.40	–	–	0.81	0.49, 1.33	1.07	0.60, 1.89
Single-feedlotZH dataset	Exposed	1.59	1.43, 1.78	<0.01	0.21	1.57	1.39, 1.78	1.70	1.30, 2.22
	Unexposed	0.53	0.42, 0.66	–	–	0.50	0.40, 0.64	0.93	0.44, 1.96

### Single-feedlot ZH Dataset

Of the 149,636 cattle at risk, 0.38% (n = 571) died during the 24-day exposure period and after accounting for potential over-dispersion in the data, the 95% CL of this estimate were 0.35% and 0.42%, respectively (Table 1). Model-adjusted estimates of the risk of death among those administered ZH and the comparative cohort were 0.48% (95% CL = 0.43, 0.53) and 0.26% (95% CL = 0.22, 0.31), respectively. After adjusting for clustering, administration of ZH was associated with an 85% increase in the risk of death (RR = 1.85 [95% CL = 1.51, 2.27]; *P*<0.01) during the at-risk period. Mortalities were classified as attributable to conditions either of the respiratory system or of the digestive system (this latter category also included all other attributable causes of death). After adjusting for clustering, administration of ZH was associated with an increase in the risk of death attributable to conditions of the respiratory (RR = 2.15 [95% CL = 1.60, 2.91]; *P*<0.01) and digestive (RR = 1.82 [95% CL = 1.31, 2.52]; *P*<0.01) systems.

Overall mortality was associated with sex and month of slaughter; in addition to their interactions with exposure, these main effects were included in a final multivariable model. The final model included sex, month and the interaction between ZH exposure and month. Averaged across other variables, animals exposed to ZH were 56% more likely to die than animals in the comparative cohort (RR = 1.56 [95% CL = 1.25, 1.95]; *P*<0.01). However, this effect was modified by the month in which animals were shipped to the abattoir (*P* = 0.01). Model-adjusted RR estimates were 3.06 (*P* = 0.01; Figure 4); 1.83 (*P* = 0.07); 2.30 (*P*<0.01); 2.42 (*P*<0.01); 1.72 (*P* = 0.01); 0.85 (*P* = 0.58), 0.80 (*P* = 0.47) and 0.97 (*P* = 0.94) for April, May, June, July, August, September, October, and November, respectively.

A number of secondary outcomes were available for analysis in this third dataset (Table 3). Animals administered ZH were 23% more likely to require treatment for any condition during the exposure period (RR = 1.23 [95% CL = 1.07, 1.41]; *P*<0.01). The likelihood of treatment, however, varied by the body system to which the clinical signs were attributed. Animals were 34% less likely to require treatment for conditions attributed to the digestive system (RR = 0.66 [95% CL = 0.55, 0.79]; *P*<0.01) but 2.3 times more likely to require treatment for conditions attributed to the respiratory system (RR = 2.31 [95% CL = 1.89, 2.83]; *P*<0.01). In addition, animals administered ZH were 2.3 times more likely to require more than a single treatment regimen for respiratory disease than contemporaneous controls (RR = 2.34 [95% CL = 1.82, 3.04]; *P*<0.01). As a consequence of the increased burden of treatments in animals administered ZH, there was a 3-fold increase in the percentage of animals subject to a slaughter withholding period at the time the rest of their group was shipped to slaughter (RR = 3.00 [95% CL = 2.31, 3.90]; *P*<0.01). Those animals that could not be shipped to slaughter with their contemporaries were dispersed across many groups rather than clustered within a few; that is, 50.0% of groups administered ZH had one or more animals that could not be shipped with the rest of their group compared to 25.8% of groups not administered ZH.

Once at the abattoir, a greater percentage of carcasses from ZH administered cattle (1.59%) were classified as dark, firm and dry (Table 4), compared to those carcasses from animals not administered ZH (0.53%; RR = 3.02 [95% CL = 2.35, 3.89]; *P*<0.01). As opposed to the 9-feedlot dataset, sex was neither a modifier of this association (*P* = 0.21) nor a significant covariate (*P* = 0.34).

## Discussion

Death is a relatively rare event in feedlot cattle [25], [26]. Even so, the data presented herein provide compelling evidence that administration of FDA-approved βAA to cattle increased both the cumulative incidence (risk) and incidence rate of death. The various measures of effect used to explore the relationship between βAA and mortality, i.e., RR, IRR and HR where applicable, were similar across the multiple datasets that included both randomized field trials and population-based observational data. The attributable fraction (AF), a measure of the proportion of deaths attributable to βAA administration among those cattle in the exposed cohort, varied little from dataset to dataset, remaining relatively constant at 40–50%.

Of considerable practical interest among those responsible for the care and well-being of cattle intended for slaughter for human consumption is identification of variables that could be exploited to help select or manage groups of cattle for which βAA drugs might be contraindicated. The most consistent modifier of the biological association between βAA administration and mortality was month of year (Figure 4). In the multi-feedlot ZH data set, month was statistically detected as an effect modifier in one model (i.e., population at-risk) but not in the other model (i.e., time at-risk). However, in the latter model, the observed *P* value (0.14) provided some, albeit weak, evidence of variation in the association across months. In the single-feedlot ZH dataset, month was again detected as a significant modifier of the association between βAA administration and mortality (*P* = 0.01). Furthermore, the consistency of the temporal pattern of this association across the multi- and single-feedlot ZH datasets (Figure 4) provides compelling reasons to explore seasonal factors that might modify the effect of βAA administration on mortality. By this we mean that month is clearly a proxy for other variables with the most probable being thermal heat index. Given the variation in the measure of effect across months, at least for ZH, it seems plausible that greater heat indices may have contributed to a disproportionate increase in the risk of death among animals administered βAA when compared to those not administered this synthetic hormone. It was not possible, however, to test this association in our analysis; clearly this warrants additional prospective exploration. If month is indeed a proxy for a causal biological interaction between some measure of heat index and βAA administration, then mitigation strategies, as yet unknown, need to be developed for a substantial proportion of the year in that a significant relationship was detected in those cattle shipped for slaughter beginning in April (in the single-feedlot ZH dataset) and for those cattle shipped for slaughter through September (in the multi-feedlot ZH dataset).

Force of mortality, which is sometimes referred to as the hazard function, is an instantaneous measure of the risk of death in the next time period, conditioned on having survived to the present. This measure was greater for those animals administered RH than unexposed animals and was relatively constant for both groups across the majority of the exposure period for company C. The apparent late-stage increase (Figure 3) in the hazard function among the RH-treated groups of animals, when compared to the control groups of animals, did not lead to a violation of the proportional hazards assumption of the semi-parametric survival analysis. However, this increase appeared quite marked and bears further examination in any follow-up research. Unfortunately, days of exposure for each animal that died were only available for company C and included 3 large randomized trials of RH of 62,379 animals. Consequently, the force of mortality for those cattle administered ZH could not be evaluated.

A number of broad hypotheses might explain the observed association of βAA drug administration and increased mortality. The first potential hypothesis is that other unmeasured confounding variable(s) gave rise to a spurious relationship. For example, in the ZH datasets, the contemporaneous control cohorts consisted of those animals not administered a βAA. There are various reasons why these animals were not fed a βAA. For example, they may have been destined for a marketing program that precluded the use of a βAA or they were deemed not suitable for βAA administration (e.g., their body weight was sufficient enough that if fed a βAA hormone, their carcass weights would have been excessive). While it is possible that these unmeasured variables confounded the association of βAA use and increased death loss, it seems highly improbable given the strength of association and consistency of effect across feedlots, datasets, and the covariates evaluated in the models. This possibility seems even more implausible in the dataset involving RH. Each of the 12 studies included randomized allocation of animals or groups to the exposed or unexposed cohorts. One of the major benefits of randomization is the unbiased distribution of unmeasured confounders among the treatment groups. Furthermore, apart from company A in which no deaths in either cohort were reported, statistical variation among the measured associations of RH administration and mortality was not detected among companies B, C, and D. In addition, no statistical variation in the association between βAA and increased death loss was observed across feedlots in the multi-feedlot ZH dataset. This association, therefore, was relatively consistent and predictable from operation to operation despite unmeasured variation in farming practices and other attributes such as feedlot size, geographical location, animal husbandry, and cattle diets.

A second and related hypothesis is that the association was not necessarily due to the drug itself; rather, the association might have been a consequence of those management changes required to administer the βAA in the ration. Such collinearity of effects is virtually impossible to disentangle without purposively designing a study (e.g., a cross-over design) to deal with the phenomenon. For example, although not available in the data described herein, it is possible that the time at which feed was delivered changed for those animals administered a βAA. That is, most modern cattle feedlots have developed strategies to provide a consistent diet in a consistent and timely manner to the cattle. In this scheme, the amount of feed delivered is expected to be consumed within 24 hours. Because a minority of groups of cattle are fed βAA at any one time in an feedlot, one management strategy might be to feed the unexposed cattle first and subsequently feed the ration containing the βAA later in the day. If so, this strategy could have resulted in a relatively sudden change in the time at which the βAA-exposed cattle were fed (possibly up to a 2-hour delay for example). While cattle tend to adapt relatively quickly to changes in routine, such a change might have initially resulted in cattle that were hungry and thus over-consumed readily fermentable carbohydrates. However, the observed force of mortality was relatively constant over the exposure period (i.e., from day 0 up to 42). The feedlots that supplied data for the 2 observational datasets have had a number of years of experience in feeding the FDA-approved βAA and have had the opportunity to develop and adopt management strategies to minimize changes in routine.

A third hypothesis is that the βAA hormones themselves are causally associated with the increased mortality. Clearly, the measure of effects are relatively strong and consistent across the datasets; yet, given that the RH studies were not purposefully designed to investigate an association with mortality and the observational nature of the ZH data, it is difficult to definitively establish a causal relationship nor to identify the mechanistic explanations. However, evidence supporting adverse drug events can be drawn from the aggregated observations of βAA administration in human medicine. For example, authors of various studies that included randomized clinical trials and an FDA-performed meta-analysis of the available data, have concluded that long-acting β_2_ adrenergic agonists used for asthma contribute to an increased risk of severe asthma events and death [8], [9], [11], [19]. While this association might be somewhat ameliorated by the inclusion of a corticosteroid, the FDA determined this was a class effect and now requires a boxed warning to be included on the labels of all long-acting βAA intended to be administered routinely, e.g., daily for the control of asthma. Furthermore, in the datasets of ZH in which risk of treatment was available, exposed animals were more likely to require treatment during the at-risk period than unexposed animals.

Others have reported an increased risk of myocardial disease in certain patients administered βAA drugs [12]. If a similar association occurs in cattle, one might expect metabolic markers of such an event, elevated creatine phosphokinase (CPK) for example [27]. Indeed, ZH administration is associated with increased serum CPK [2]; unfortunately the isoform(s) were not reported so it is uncertain if the elevated serum CPK resulted from damage to striated muscle, myocardial tissue, or both.

After accounting for the effect of exposure, most of the unexplained variation in mortality occurred at the level of the group rather than at the organizational levels of the study, feedlot, or company depending on the dataset. Moreover, there was almost no (and at times zero) unexplained variation attributed to unmeasured company or feedlot level factors. In other words, the biological association did not vary sufficiently among companies or feedlots to be detected by the statistical methods described herein. Group-level factors, therefore, seem to be important determinants (or modifiers) of mortality and if discovered, may influence the design of management strategies to reduce mortality, particularly in pens administered βAA. Unfortunately, we could not identify any meaningful and consistent effect modifiers, other than month, in the data and further research is needed to identify factors that may modify the observed association between βAA and increased risk of death. Saliently, the statistical variation of groups is a function of both the individuals as well as the interaction among the individuals within the group. In addition to group-level management strategies, therefore, 2 additional lines of investigation ought to be pursued and include animal-level factors, such as genetic variation in response to βAA [28], and how individuals interact to influence the behavior and potentially the response to βAA of other animals.

The data reported have considerable strengths in that the number of observations permitted the detection of a change in a rare event (or events, if one considers the secondary outcomes). When seeking approval for products, it is extremely uncommon to have available such numbers to detect rare adverse drug events. Based on the results observed herein, the number needed to harm, a measure of extent of exposure required for a βAA-related death, was approximately 500 animals or 15,000 animal days. In most reports of well-controlled cattle experiments, the number of animals included is usually insufficient to speak to an association of βAA with mortality, given the rarity of death and estimates of the number needed to harm. As a result, most drug approvals require some post-approval monitoring, often termed the pharmaco-epidemiology or Type IV trials, and the self-reporting by patients of side-effects, either to the company, the FDA, or their doctor, is part and parcel of a holistic drug regulatory framework. Furthermore, our data are from commercial cattle fattening feedlots and as such, provide a degree of validity to the results. However, the number of companies and feedlots is relatively limited and consequently, selection bias is possible in that the decision to provide the data for inclusion in the analyses may have been influenced by a suspicion of increased mortality associated with βAA administration.

Despite the potential limitations of the data, we argue that given the magnitude of the data, and the strength and consistency of the various measures of effect, both RH and ZH are most likely causally associated with increased cumulative incidence, incidence rate, and hazard of death when they are administered in accordance with the FDA-approved label directions. The excess deaths attributed to βAA administration, and potentially the secondary outcomes of illness and occurrence of beef classified as *dark, firm and dry*, represent adverse drug events. If so, we believe a broad and inclusive dialogue that explores the balance between improved production efficiencies achieved through means such as βAA [29] and resultant adverse effects on the welfare of animals we raise for food is needed. This is particularly warranted for those drugs that are approved solely to improve the efficiencies of production yet offer no offsetting health benefits to the animals to which it is administered. For this dialogue to be sufficiently inclusive, it ought to include a broad collection of stakeholders such as animal scientists, cattle and beef producers, animal health specialists, welfarists, ethologists, and consumers.
